# Knowledge, attitudes, and practices of healthcare providers toward ADR reporting regarding thiopurine drugs for the treatment of acute lymphoblastic leukemia at the University of Gondar Comprehensive Specialized Hospital, Northwest Ethiopia

**DOI:** 10.3389/fphar.2025.1486415

**Published:** 2025-05-19

**Authors:** Eyuel Kassa, Mastewal Birhan, Mulugeta Ayalew, Aschalew Gelaw, Abdulkader Mohamedsaid Gidey, Degalem Tilahun Worku, Awole Mekonen, Ermias Teklehaimanot, Assefa Asnakew, Alazar Amare, Nega Berhane Tessemma

**Affiliations:** ^1^ Department of Medical Biotechnology, Institute of Biotechnology, University of Gondar, Gondar, Ethiopia; ^2^ College of Veterinary Medicine and Animal Sciences, University of Gondar, Gondar, Ethiopia; ^3^ Unit of Pediatric Hematology Oncology, Department of Pediatrics and Child Health, School of Medicine, College of Medicine and Health Sciences, University of Gondar, Gondar, Ethiopia; ^4^ Department of Medical Microbiology, School of Biomedical and Laboratory Sciences, College of Medicine and Health Sciences, University of Gondar, Gondar, Ethiopia; ^5^ Unit of Pediatric Hematology Oncology, Department of Pediatrics and Child Health, School of Medicine, College of Medicine and Health Sciences, Addis Ababa University, Addis Ababa, Ethiopia; ^6^ Department of pathology, School of Medicine, College of Medicine and Health Sciences, University of Gondar, Gondar, Ethiopia; ^7^ Department of Pharmacy, Asrat Woldeyes Health Science Campus, Debre Berhan University, Debre Berhan, Ethiopia; ^8^ Unit of Molecular Biology and Bioinformatics, Institute of Biotechnology, Addis Ababa University, Addis Ababa, Ethiopia; ^9^ Medical Laboratory Department, Dilla University College of Medicine and Health Sciences, Dilla, Ethiopia

**Keywords:** knowledge, attitude, practice, acute lymphoblastic leukemia, thiopurine drugs, adverse drug reactions, thiopurine methyltransferase gene

## Abstract

**Background:**

Knowledge, attitudes, and practices (KAP) regarding the use of thiopurine chemotherapeutic drugs in the treatment of acute lymphoblastic leukemia (ALL) are critical for healthcare professionals. Thiopurines are associated with varying levels of toxicity, including myelosuppression, hepatotoxicity, and gastrointestinal intolerance. Approximately 20% of ALL patients discontinue thiopurine therapy due to toxicity and other adverse events. This study aims to assess the KAP of healthcare providers (HCPs) concerning thiopurine drugs used in the treatment of ALL in hospital wards in Northwestern Ethiopia.

**Methods:**

A hospital-based cross-sectional study was conducted among 161 HCPs at the University of Gondar Comprehensive Specialized Hospital from June 1, 2023, to August 30, 2023. Data were collected using a self-administered questionnaire. The collected data were coded, entered and analyzed using SPSS version 25. Associations between categorical variables were assessed using cross tabulation, and both crude and adjusted odds ratios were calculated with a 95% confidence interval. Variables with *p*-values less than 0.05 were considered statistically significant.

**Results:**

A total of 161 HCPs participated in the study, comprising 69 (42.9%) females and 92 (57.1%) males. The majority of participants were nurses 105 (65%), followed by physicians 20 (12.4%), pharmacists 19 (11.8%), midwives 10 (6.2%), and health officers 7 (4.3%). More than half of the participants demonstrated inadequate knowledge 94 (58.4%), negative attitudes 82 (50.9%), and poor practices 90 (55.9%) regarding the use of thiopurine drugs for the treatment of ALL. Approximately 50 (31.1%) participants understood the term “thiopurine drugs,” while 89 (55.3%) were aware of their adverse drug reactions. However, the majority 136 (84.5%) had not received training on how to report adverse drug reactions.

**Conclusion:**

The majority of healthcare professionals demonstrated negative attitudes, inadequate knowledge, and poor practices concerning the reporting of adverse drug reactions associated with thiopurine drugs.

## Introduction

Acute lymphoblastic leukemia (ALL) is the most common childhood cancer worldwide. Treatment outcomes have significantly improved over the past several decades due to the combined use of multiple chemotherapeutic agents and advanced therapeutic protocols (Duffield et al., 2023). Thiopurines, such as thioguanine, 6-mercaptopurine, and azathioprine, are widely used in clinical practice for the treatment of children with ALL and inflammatory bowel diseases. However, these drugs are associated with severe adverse drug reactions (ADRs), including allergic reactions (25%), liver test abnormalities (34%), nausea and vomiting (6%), bone marrow suppression (7%), hepatotoxicity, gastrointestinal intolerance, myelosuppression, and secondary tumor formation. These serious ADRs pose significant challenges in reducing ALL-related mortality and limit the broader application of thiopurines (Gearry et al., 2004; Chan and Ismail, 2014; Bárcenas-López et al., 2021; Zhu et al., 2018).

Metabolizing enzymes, such as thiopurine S-methyltransferase (TPMT), and drug transporters, including multidrug resistance-associated proteins, have been reported to play a critical role in the metabolism and transportation of thiopurine drugs (Guo et al., 2022). Genetic variations in drug-metabolizing enzymes and drug transport systems can lead to significant interindividual differences in drug exposure, resulting in toxicity in a substantial proportion of patients (Sileshi et al., 2022; Shyamveer and Singh, 2023). Consequently, patients undergoing thiopurine therapy require routine monitoring of blood cell counts. Thiopurines are typically administered daily as an oral dose for up to two and a half years during the maintenance phase of therapy (Toksvang et al., 2022). Pharmacogenetic factors contributing to thiopurine-induced myelotoxicity are partially attributed to genetic variants in the *TPMT* gene (Franca et al., 2019).

Today, with the use of intensive multi-agent chemotherapy, the majority of children with ALL can be cured, with overall remission rates typically exceeding 90% (Ramadhan et al., 2024). However, childhood cancer survival rates are significantly lower in low-income countries (ranging from 5% to 60%) compared to high-income countries (approximately 90%) (Farrag et al., 2023). This disparity can be attributed to several factors, including death from toxicity, relapse, and non-adherence to treatment. Chemotherapy aims to achieve three primary goals: cure, control, or palliation (Neugut and Prigerson, 2017; El Kamar et al., 2003). Nevertheless, chemotherapy-related side effects can adversely affect a patient’s quality of life and hinder families’ acceptance of and adherence to prescribed medications. Non-adherence to cancer treatment is associated with an increased risk of relapse and reduced survival rates. Healthcare providers (HCPs) perceptions of drug side effects and toxicity can influence their attitudes and management strategies, which in turn may impact patient adherence and treatment outcomes (Handayani et al., 2022).

Globally, thousands of drugs enter the market daily; however, medication safety remains a significant concern for diverse populations due to insufficient knowledge. According to the World Health Organization (WHO), an ADR is defined as any harmful, unintended, or undesired response to a drug that occurs at therapeutic doses used for prevention, diagnosis, or treatment, or for the modification of physiological functions. This definition excludes reactions resulting from accidental or intentional overdoses or medication errors (Seid et al., 2018). Adverse drug reactions can be categorized as predictable and dose-related, unpredictable and non-dose-related, dose- and time-related (delayed reactions), withdrawal reactions, or unexpected reactions due to treatment failure (Kassa Alemu and Biru, 2019). Pharmacovigilance encompasses the science and activities related to detecting, assessing, understanding, and preventing adverse effects or any other potential drug-related problems (Kassa Alemu and Biru, 2019; Chatterjee and Aparasu, 2023; Cervantes-Arellano et al., 2024).

Nearly half of all medicines worldwide are used irrationally, leading to severe consequences such as **ADRs,** drug toxicity, drug resistance, prolonged illness, and even death (Melku et al., 2021). The underreporting of ADRs associated with thiopurine and other chemotherapy drugs remains a persistent challenge in Ethiopia. This study assessed **HCPs knowledge and attitudes** toward ALL patients and their chemotherapy treatments. The findings reveal significant gaps in understanding among healthcare professionals, which directly impacts patient care. These insights are valuable for improving the quality of treatment for childhood leukemia. Furthermore, this information can guide the development of more effective patient education strategies and serve as a foundation for future research by HCPs. By identifying specific gaps in knowledge, attitudes, and practices, this study contributes to the broader goal of optimizing chemotherapy management in resource-limited settings.

## Methods

### Study area, design, and period

A hospital-based cross-sectional study was conducted from June 1, 2023, to August 30, 2023, among healthcare providers (HCPs) working at the UoGCSH. The hospital is located in the Amhara National Regional State (ANRS), Northwestern Ethiopia, approximately 750 km from Addis Ababa. It serves a population of over 7 million people in its catchment areas and a wide range of health services, including pediatric hematology and oncology services. Notably, the pediatric hematology-oncology center at UoGCSH is the only facility of its kind in the ANRS (Taddese et al., 2020; Tesfay et al., 2022).

### Study population

The study population included all physicians, pharmacists, nurses, health officers, and midwives who were working in various pediatric units at the UoGCSH during the study period. The units comprised Pediatric Oncology Ward (*N* = 10), Neonatology Ward (*N* = 38), Main Pediatric Wards (*N* = 10), Pediatric Surgical and Emergency Ward (*N* = 11), Pediatric Emergency Ward (*N* = 20), Pediatric ICU (*N* = 8), Maternity Ward (*N* = 30), Pediatric Ward Pharmacy (*N* = 20), and Pediatric outpatient department (OPD) (*N* = 7). Additionally, healthcare providers from the adolescent oncology ward (*N* = 10) were included in the study.

### Inclusion and exclusion criteria

Healthcare providers were eligible for inclusion in the study if they were current employees of the UoGCSH and held a valid hospital identification card. Participants were also required to fall within the age intervals 18–60 years. Furthermore, they needed to have earned a diploma, degree, or master’s qualification from a recognized university accredited by the Ethiopian Ministry of Health and the Ministry of Education including private organization. Healthcare providers who declined to participate, who were on leave, or were absent from work during the data collection period due to illness or other reasons were excluded from the study.

### Dependent and independent variables

The study identified the knowledge, attitudes, and practices (KAP) of HCPs regarding the use of thiopurine drugs for the treatment of ALL as the dependent variables. The independent variables included age, sex, profession, level of education, years of experience, and training on ADR reporting related to chemotherapy.

### Operational definition and measurements

• **Adverse Drug Reactions (ADRs):** ADRs are defined as any noxious, unintended, or undesired effects of a drug that occur at doses typically used in humans for prophylaxis, diagnosis, or therapy (Zimamu et al., 2021).
• **Side Effects:** Side effects refer to unintended effects that occur at normal doses and are related to the pharmacological properties of the drugs.
• **Pharmacovigilance:** Pharmacovigilance encompasses the science and activities related to the detection, assessment, understanding, and prevention of ADRs or any other medicine-related problems, aiming to improve the safety of medicines (Zimamu et al., 2021).
• **Knowledge:** Knowledge refers to the information stored in memory and was assessed based on participants’ understanding of thiopurine drugs and their adverse effects (Zimamu et al., 2021).
• **Adequate Knowledge:** Participants who correctly answered ≥50% of the 22 knowledge-related questions. Each correct answer was scored 1, and incorrect answers were scored 0.
• **Inadequate Knowledge:** Participants who answered <50% of the knowledge-related questions about thiopurine ADRs.
• **Attitude:** Attitude refers to the complex interaction of beliefs, feelings, and values that influence responses to thiopurine ADRs and their adverse effects (Zimamu et al., 2021).
• **Good Attitude:** Participants who answered ≥50% of the attitude-related questions about thiopurine ADRs correctly.
• **Poor Attitude:** Participants who answered <50% of the attitude-related questions about thiopurine ADRs in the context of ALL treatment reporting.
• **Practice:** Practice refers to the actual application or use of an idea, belief, or method, as opposed to theories related to it (Zimamu et al., 2021).
• **Good Practice:** Participants who answered ≥50% of the practice-related questions about thiopurine ADRs for ALL treatment reporting correctly.
• **Poor Practice:** Participants who answered <50% of the practice-related questions about thiopurine ADRs for ALL treatment reporting

### Sampling technique and sample size determination

The study included all HCPs working in various pediatric units, comprising 178 physicians, nurses, pharmacies, health officers, and midwives who were directly or indirectly involved in the management of thiopurine drug treatments were included in the study. Since the entire study population was included, the sampling technique used was the census methodology.

### Data collection tool and data collection process

Data were collected using a structured questionnaire developed through an extensive review of the literature. The self-administered questionnaire was designed to gather relevant information on sociodemographic characteristics and the assessment of KAP. The questionnaire was adapted from tools used in similar studies and aligned with the guidelines provided by the Ethiopian Food and Drug Administration (EFDA) (Seid et al., 2018; Kassa Alemu and Biru, 2019). Prior to the commencement of the study, a pretest was conducted. The questionnaire, along with an attached written consent form, was distributed to the HCPs.

In this survey, the knowledge of HCPs regarding thiopurine ADRs for the treatment of ALL was assessed using 22 questions. Each correct response was assigned a score of one (1), while each incorrect response was scored as zero (0), resulting in a total score ranging from 0 to 22 points. The overall level of knowledge was categorized based on the mean score. Participants who scored equal to or above the mean were classified as having adequate knowledge, whereas those scoring below the mean were classified as having inadequate knowledge.

Participants’ attitudes were evaluated using 14 questions rated on a three-point Likert scale (agree, neutral, and disagree). An “agree” response was assigned a score of 3, a “neutral” response a score of 2, and a “disagree” response a score of 1. A score equal to or greater than 50% indicated a good attitude, while a score below 50% indicated a poor attitude toward thiopurine ADRs in the treatment of ALL.

Healthcare providers were further assessed based on whether they documented and reported ADRs. A correct response required not only providing the precise definition but also demonstrating a general understanding of pharmacovigilance concepts.

### Data quality control measures

Following the preparation of the data collection format, a pretest of the tool was conducted with 10 HCPs working at the UoGCSH. This pretest aimed to identify any necessary amendments and ensure the tool’s suitability for actual data collection. The collected data were reviewed and checked daily for completeness and consistency before proceeding with data processing and analysis.

### Data analysis

The data were entered into Epi Data version 4.3, cleaned, and subsequently exported to the Statistical Package for Social Sciences version 25 (SPSS-25) for further analysis. A binary logistic regression model was employed to assess the relationship between the outcome variable (knowledge, attitudes, and practices [KAP] status of HCPs and predictor variables (sociodemographic factors). The association between selected categorical variables was examined using cross-tabulation, and Pearson chi-square tests were conducted. Crude and adjusted odds ratios were calculated with 95% confidence intervals. Variables with a p-value of less than 0.05 were considered statistically significant.

## Results

### Sociodemographic characteristics

A total of 178 healthcare providers were approached for the study. Of these, 161 participants completed the self-administered questionnaire correctly and returned it within the stipulated time, yielding a response rate of 90.4%. The majority of the healthcare providers, 92 (57.1%), were male. In terms of age distribution, 109 (67.7%) participants were between 26 and 35 years old. The largest professional group among the respondents was nurses, comprising 105 (65.2%) of the total, followed by physicians (20, 12.4%), pharmacists (19, 11.8%), health officers (7, 4.3%), and midwives (10, 6.2%). Approximately 132 respondents (82.0%) reported having more than 3 years of professional experience (Table 1).

**TABLE 1:** Sociodemographic characteristics of healthcare providers at the University Gondar Comprehensive Specialized Hospital Northwestern Ethiopia, 2023 (*n* = 161).

Variables	Category	Frequency (*N*)	Percentage (%)
Gender	Female	69	42.9
	Male	92	57.1
Age range in years	18–25	20	12.4
	26–35	109	67.7
	36–45	27	16.8
	46–55	4	2.5
	56–65	1	0.6
Profession	Physicians	20	12.4
	Pharmacy	19	11.8
	Nurse	105	65.2
	Health officer	7	4.3
	Midwives	10	6.2
Level of education	Diploma	4	2.5
	Degree	118	73.3
	MSc	22	13.7
	Specialist	2	1.2
	MD	15	9.3
Year of clinical experience	≤3 year	29	18
	>3 year	132	82

N, Number of study participants; MSc, Master Of Science, MD, Medical doctors.

### Knowledge of healthcare providers in the treatment of acute lymphoblastic leukemia

Twenty-two questions were used to assess HCPs knowledge of the treatment of ALL patients. More than half of the respondents, 81 (50.3%), correctly identified ALL as the most prevalent childhood blood cancer, as opposed to acute myeloid leukemia (AML) (Table 2). The majority of HCPs, 116 (72.0%), correctly identified ALL as a cancer of the white blood cells (WBC). However, less than half of the respondents, 68 (42.2%), were aware that ALL is more common in children than in adults. Additionally, only a small proportion of HCPs, 25 (15.5%), knew that ALL has a cure rate of 98%. Conversely, a significantly higher number of respondents, 125 (79.5%), incorrectly believed that chemotherapy only makes children sicker and cannot cure cancer. Furthermore, 15 respondents (9.3%) incorrectly believed that blood cancer can be prevented by vaccination.

**TABLE 2:** Knowledge of HCPs on the treatment of acute lymphoblastic leukemia at the UoGCSH, Northwestern Ethiopia, 2023 (*n* = 161).

Items	Category	Profession							
		Physicians	Pharmacy	Nurse	Health officers	Midwives	Total (f)	Pearson chi-square	*p*-value
		20(12.4%)	19 (11. 8%)	105 (65.2%)	7 (4.3%)	10 (6.2%)	161 (100%)		
Have you heard about the term thiopurine drugs?								34.266	0.000
Yes		14 (28)	12 (24)	24 (48)	0 (0)	0 (0)	50 (31.1)		
No		6 (5.4)	7 (6.3)	81 (73.0)	7 6.3)	10 (9.0)	111 (68.9)		
If yes for Q1, from where did you get the information?								62.694	0.000
Internet		1 (1.6)	5 (7.9)	46 (73.0)	4(6.3)	7 (11.1)	63 (39.1)		
Colleagues		4 (6.0)	6 (9.0)	51 (76.1)	3(4.5)	3(4.5	67 (41.6)		
Formal education		15 (48.40	8 (25.8)	8 (25.8)	0	0	31 (19.3		
Which diseases do you think that thiopurine drugs are prescribed?								33.877	0 .001
Liver cancer		0	4 (13.3)	22 (73.3)	0	4 (13.3)	30 (18.6)		
ALL		13 (14.8)	14 (15.9)	58 (65.9)	0	3 (3.4)	88 (54.7)		
CML		0	0	1 (100)	0	0	1 (0.6)		
I don’t know		7 (16.7)	1(2.4)	24 (57.1)	7 (16.7)	3 (7.1)	42 (26.1)		
Do you think that thiopurine drugs have adverse reactions?								28.452	0.000
Yes		14(15.7)	16 (18.0)	55 (61.8)	1(1.1)	3 (3.4)	89 (55.3)		
No		2 (4.5)	3 (6.8)	33 (75.0)	1(2.3)	5 (11.4)	44 (27.3)		
I don’t know		4 (14.3)	0	17 (60.7)	5(17.9)	2 (7.10	28 (17.4)		
If yes for Q4, which one is the adverse effect of thiopurine drugs?								68.757	0.000
Mylosupression		3 (9.45)	5 (15.6%)	21 (65.6%)	0	3 (9.4)	32 (19.9)		
Leukopenia		0	4 (8.9%)	31 (68.9%)	6 (13.3%)	4 (8.9%)	45 (28.0)		
Hepatotoxicity		2 (4.0%)	2 (4.05)	43 (86.0%)	0	3 (6.0%)	50 (31.1)		
All		8 (44.1%)	8 (23.1%)	10 (29.4%)	1(2.9%)	0	34 (21.1)		
Which is more prevalent in childhood blood cancer?								6.239	0.182
AML		8 (10.0%)	11 (13.8%)	51 (63.8%)	2 (2.5%)	8 (10.0%)	80 (49.7)		
ALL		12(14.8%)	8 (9.9%)	54 (66.7%)	5 (6.2%)	2 (2.5%)	81 (50.3)		
Have you trained on how to report ADR?								22.967	0.000
Yes		10(40.0%)	4 (16.0%)	10 (40%)	0	1 (4.0%)	25 (15.5)		
No		10 (7.4%)	15(11.0%0	95(69.9%)	7(5.1%)	9 (6.6%)	136 (84.5)		
Have you heard of the term TPMT gene								27.454	0.000
Yes		10(45.5%)	3 (11.5%)	9 (40.9%)	0	0	22 (13.7)		
No		10 (7.2%)	16 (11.5%0	96 (69.1%)	7 (5.0%)	10 (7.2%)	139 (86.3)		
Have you heard of the term TPMT enzyme deficient?								11.344	0.023
Yes		7 (31.8%)	4 (18.2%)	10 (5.5%)	0	1 (4.5%)	22 (13.7)		
No		13 (9.4%)	15 (10.8%)	95(68.3%)	7(5.0%)	9 (6.5%)	139 (86.3)		
Does chemotherapy make children only sicker and therefore cannot cure cancer?								33.754	0.440
Yes		6 (18.2%)	6 (18.2%)	19 (7.6%)	1(3.0%)	1 (3.0%)	33 (20.5)		
No		14 (10.9%)	13 (10.2%)	86 (67.2%)	6(4.7%)	9 (7.0)	128 (79.5)		
Knowing the term pharmacovigilance?								35.982	0.000
Yes		13 (35.1%)	9 (24.3%)	15 (40.5%)	0	0	37 (23.0)		
No		7 (5.6%)	10 (8.1%)	90 (72.6%)	7(5.6%)	10 (8.1%)	124 (77.0)		
Knowing national ADR reporting system?								27.407	0.000
Yes		12(35.3%)	7 (20.6%)	14 (41.2%)	0	1 (2.9%)	34 (21.1)		
No		8 (6.3%)	12 (9.4%)	91 (71.7%)	7(5.5%)	9 (7.1%)	127 (78.9)		
Knowing availability of ADR reporting forms?								30.228	0.000
Ye		12(37.5%)	7 (21.9%)	11 (34.4%)	1 (3.1%)	1 (3.1%)	32 (19.9)		
No		8 (6.2%)	12 (9.3%)	94 (72.9%)	6 (4.7%)	9 (7.0%)	129 (80.1)		
Knowing the responsible body that monitors ADR in Ethiopia?								32.376	0.000
Ye		12(36.4%)	8 (24.2%)	12 (6.4%)	0	1 (3.0%)	33 (20.5)		
No		8 (6.2%)	11 (8.6%)	93 (2.7%)	7 (5.5%)	9 (7.0%)	128 (79.5)		
Knowing ADR reporting a professional obligation?								38.568	0.000
Yes		15(23.8%)	12 (19.0%)	29 (6.4%)	7(11.1%)	0	63		
No		5 (5.1%)	7 (7.1%)	76 (7.6%)	0	10 (10.0%)	98		
Which medications are appropriate for ALL types of blood cancers?								18.157	0.111
Mercaptopurin		13(20.6%)	9 (14.3%)	37 (58.7%)	1 (1.6%)	3 (4.8%)	63 (39.1)		
Thioguanine		4 (8.5)	6 (12.8%)	32 (68.1%)	3 (6.4%)	2 (4.3%)	47 (29.2)		
Azathiopurine		2 (5.9%)	3 (8.8%)	21 (61.8%)	3(8.8%)	5 (14.7%)	34 (21.1)		
I don’t know		1 (5.9%)	1 (5, 9%)	15(88.2%)	0	0	17 (10.6)		
Testing strategies to access TPMT activity?								6.742	0.565
Phenotype		4 (18.2%)	4 (18.2%)	13(59.1%)	1(4.5%)	0	22 (13.7)		
Genotype		8 (12.7%)	9 (14.3%)	41(65.1%)	1(1.6%)	4 (6.3%)	63 (39.1)		
WBC		8 (10.5%)	6 (7.9%)	51(67.1%)	5(6.6%)	6 (7.9%)	76 (47.2)		
TPMT Genotype test is not affected by?								13.949	0.304
Blood transfusions		7 (14.3%)	8(16.3)	28(57.1%)	3 (6.1%)	3 (6.1%)	49 (30.4)		
Concomitant medications		3 (7.9%)	6(15.8%)	27(71.1%)	2 (5.3%)	0	38 (23.6)		
All		9 (13.0%)	5(7.2%)	47(68.1%)	1 (1.4%)	7(10.1%)	69 (42.9)		
I don’t know		1 (20.0%)	0	3 (60.0%)	1 (20.0%0	0	5 (3.1)		
Acute lymphoblastic leukemia is?								11.966	0.153
Cancer in WBC		16(13.8%)	12 (10.3%)	77(66.4%)	6 (4.3%)	6 (5.2%)	116 (72.0)		
Cancers in RBC		2 (8.0%)	6 (24.0%)	12(48.0%)	1 (4.0%)	4 (16.0%)	25 (15.5)		
Cancer in LN		2 (10.0%)	1 (5.0%0	16 (80%)	1 (5.0)	0	20 (12.4)		
Can blood cancer be prevented through Vaccination?								10.146	0.255
Yes		6 (36.7%)	7 (40.0%)	18 (1.3%)	2 (10%)	2 (10%)	35 (21.7)		
No		14 (11.1%)	12 (9.5%)	87 (9.0%)	5 (4.0%)	8 (6.3%)	126 (78.3)		
Which of the following subgroups of the populations are more prone to catching or an increased risk of ALL?								43.088	0.002
Pregnant females		1 (4.0%)	3 (12.0%)	16 (4.0%)	1 (4.0%)	4 (16.0%)	25 (15.5)		
Elderly		0	4 (12.9%)	19 (1.3%)	5 (16.1%)	3 (9.7%)	31 (19.3)		
Children		17(25.0%)	8 (11.8%)	42 (1.8%)	0	1 (1.5%)	68 (42.2)		
Adults		1 (7.7%)	2 (15.4%)	8 (61.5%)	0	2 (15%0	13 (8.1)		
All		1 (4.8%)	1 (4.8%)	18 (5.7%)	1(4.8%)	0	21 (13.0)		
I don’t know		0	1 (33.3%)	2 (66.7%)	0	0	3 (1.9)		
Is ALL curable? A. Yes (n = 64), B. No (n = 97), if you say yes, at what percentage?								21.716	0.153
98%		6 (24.0%)	3 (12.0%)	16 (4.0%)	0	0	25 (15.5)		
50%		3 (6.2%)	5 (10.4%)	34 (0.8%)	2 (4.2%)	4 (8.3%)	48 (29.8)		
25%		6 (19.4%)	5 (16.1%)	17 (4.8%)	3 (9.7%)	0	31 (19.3)		
4%		2 (0.5%)	1 (5.3%)	11 (9.9%)	2(10.5%)	3(15.8%)	19 (11.8)		
I don’t know		3 (7.9%)	5 (13.2%)	27 (1.1%)	0	3 (7.9%)	38 (23.6)		

The association was assessed using the Pearson chi-square test, with statistical significance indicated by *p* < 0.05. UoGCSH, University of Gondar Compressive Specialized Hospital; ADR, adverse drug reaction; TPMT, thiopurine methyltransferase; ALL, acute lymphoblastic leukemia; WBC, white blood cell; RBC, red blood cell; LN, lymph node; HCPs, healthcare providers; CML, chronic myelogenous leukemia.

A considerable proportion of HCPs were unfamiliar with key terms related to thiopurine drugs and their mechanisms: 111 (68.9%) were unaware of “thiopurine drugs,” 139 (86.3%) were unfamiliar with the TPMT gene, 76 (47.2%) did not know about TPMT enzyme deficiency, and 69 (42.9%) were unaware of “testing strategies to assess TPMT activity.” Nevertheless, a majority of respondents, 89 (55.3%), correctly recognized thiopurine ADRs, although only 34 (21.1%) knew that these ADRs include myelosuppression, leukopenia, or hepatotoxicity. Additionally, 63 (39.1%) of the HCPs correctly identified mercaptopurine (6-MP) as the drug of choice for ALL. In contrast, 47 (29.2%) believed azathioprine and 24 (21.1%) thought thioguanine were the drugs of choice for ALL, indicating some misconceptions among respondents.

Among the 161 study participants, less than half, 63 (39.1%), knew that ADR reporting is a professional obligation. Most respondents, 136 (84.5%), had not received training on ADR reporting. Additionally, only 33 (20.5%) of HCPs were able to identify the responsible body for monitoring ADRs in Ethiopia. The majority of respondents, 124 (77.0%), were unfamiliar with pharmacovigilance and could not accurately define it. Physicians, compared to other healthcare professionals, demonstrated significantly higher knowledge about pharmacovigilance (65.0%, *p* < 0.05).

In the overall assessment of knowledge, most participants answered questions incorrectly regarding ADR types and treatments for ALL, with an error rate exceeding 94 (58.4%). Among the 161 respondents, physicians (60.0%, *p* < 0.05) and pharmacists (36.8%, *p* < 0.05) were significantly more aware of the national ADR reporting system in Ethiopia. Physicians (76.45%, *p* < 0.05) also demonstrated significantly greater awareness of the availability of ADR reporting forms compared to other professionals. However, 129 respondents (80.1%) were unaware of the existence of the national ADR reporting form, which hindered their ability to report ADRs. Similarly, 136 (84.5%) respondents were uncertain about how to report ADRs, further impeding their reporting practices. Additionally, 128 (79.5%) healthcare professionals refrained from reporting ADRs due to the absence of a responsible body monitoring ADRs in Ethiopia (Table 2).

Regarding methods for obtaining information on thiopurine drug reporting, respondents indicated awareness of at least one method, such as reporting via the internet (39.1%), through colleagues (41.6%), or via formal education (19.3%). However, 127 participants (78.9%) did not know any methods for ADR reporting. Some participants also selected multiple answers regarding methods used for ADR reporting (Table 2).

### Association of years of experience with knowledge of ADR reporting

According to the findings of this study, healthcare professionals with more than 3 years of experience demonstrated significantly higher awareness of the national ADR reporting system (80%, *p* < 0.05). In relation to the availability of the ADR reporting form, healthcare professionals with less than 3 years of experience showed a lower awareness level at 12.5% (*p* = 0.335), whereas those with more than 3 years of experience exhibited a significantly higher awareness level at 87.5% (*p* < 0.05).

### Knowledge and general awareness of ADR reporting among healthcare providers

The general awareness of respondents regarding ADR reporting, more than half of the respondents, 80 (49.7%), indicated that they obtained information about ADRs from the National Drug Formulary and Standard Treatment Guidelines (STGs), followed by standard textbooks, 18 (11.2%). Regarding ADR reporting practices, responses varied significantly: 43 (26.7%) mentioned reporting to manufacturers, 37 (23.9%) to the Ministry of Health (MOH), 21 (13.9%) to pharmacy departments, 13 (8.1%) to the Ethiopian Pharmaceutical Association (EPA), 15 (9.3%) to the Ethiopian Food and Drug Administration (EFDA), and 16 (9.9%) to the Drug and Therapeutic Committee (DTC) of their respective health facilities.

Factors reported to increase the likelihood of ADRs among patients included overdose, prescription errors, dispensing errors, patient lifestyle, and non-adherence. These factors were reported by 52 (32.3%), 27 (16.8%), 22 (13.7%), 22 (13.7%), and 8 (5.0%) of respondents, respectively. Notably, 26 (16.5%) identified both prescribing and dispensing errors as predisposing factors. However, only a few respondents, 4 (2.5%), correctly identified all these factors as contributing to ADRs (Table 3).

**TABLE 3:** General awareness of HCPs about ADR reporting at the UoGCSH, Northwestern Ethiopia, 2023 (*n* = 161).

Items	Category	Profession							
		Physicians	Pharmacy	Nurse	Health officer	Midwives	Total	Pearson chi-square	*p*-value
		20 (12.4%)	19 (11. 8%)	105 (65.2%)	7 (4.3%)	10 (6.2%)	161 (100%)		
To whom do you think ADRs should be reported?								50.791	0.005
Manufacturers		5 (11.6)	2 (4.7)	31 (72.1)	4 (9.3)	1 (2.3)	43 (26.7)		
MOH		3 (8.1)	4 (10.8)	26 (70.3)	1(2.70	3 (8.1)	37 (23.0)		
EPA		1 (7.70	0	9 (69.20	0	3 (23.10	13 (8.1)		
EFDA		19 (6.7)	3(20.0)	9 (60.0)	1(6.7)	1 (6.7)	15 (9.3)		
DTC of the respective health facility		2 (12.5)	2 (12.5)	12 (75.0)	0	0	16 (9.9)		
Pharmacy department		0	5 (23.8)	13 (61.9)	1 (4.8)	2 (9.5)	21 (13.0)		
all		8 (53.3)	3 (20.0)	4 (26.7)	0	0	15 (9.3)		
I do not know		0	0	1 (100)	0	0	1(0.6)		
Who is primarily responsible for reminding and follow-up patients about the side effects of drugs they are given?								30.065,	0.026
Physician		8 (12.1)	4 (6.1)	47 (71.2)	4 (6.1)	3 (4.5)	66 (41.0)		
Pharmacy		4 (10.3)	6 (15.4)	23 (59.0)	3 (7.7)	3 (7.7)	39 (24.2)		
Health officer		0	1 (11.1)	6 (66.7)	0	2 (22.2)	9 (5.6)		
Nurses		0	2 (8.7)	19 (82.6)	0	2 (8.70	23 (14.3)		
Midwifery		0	0	1 (100)	0	0	1 (0.6)		
I do not know		8 (34.8)	6 (26.1)	9 (39.1)	0	0	23 (14.3)		
What is your source of information about ADR?								39.586	0.313
National drug formulary and STG		17 (21.2)	13 (16.2)	41 (51.2)	5 (6.2)	4 (5.0)	80 (49.7)		
Standard text books		1 (5.6)	1 (5.60	13 (72.20	1 (5.6)	2 (11.1)	18 (11.2)		
Drugs sales man		1 (6.7)	2 (13.3)	12 (80.0)	0	0	15 (9.3)		
Notes from the training		0	0	8 (80.0)	0	2 (20.0)	10 (6.2)		
Search engines (Internet)		0	1 (6.2)	15 (93.8)	0	0	16 (9.9)		
Journal article		1(12.5)	1 (12.5)	5 (62.5)	0	1 (12.5)	8 (5.0)		
Package inserts		0	0	5 (100)	0	0	5 (3.1)		
Advertisement brochures/leaflets		0	0	3 (75.0)	0	1(25.0)	4 (2.5)		
Direct call to a pharmaceutical company		0	0	1 (100)	0	0	1 (0.6)		
Pharmaceutical company representative		0	1 (25.0)	2 (50)	1 (25.0)	0	4 (2.5)		
What possible factor (s) predisposes a patient to ADR?								59.973	0.000
Dispensing error (1)		2 (9.1)	1 (4.5)	14 (63.6)	3 (13.6)	2 (9.1)	22 (13.7)		
Prescription error (2)		1 (3.7)	3 (11.1)	18 (66.7)	1 (3.7)	4 (14.8)	27 (16.8)		
Overdose		5 (9.6)	5 (9.6)	37 (71.2)	2 (3.8)	3 (5.8)	52 (32.3)		
The life style of the patient		1 (4.5)	1(4.5)	19 (86.4)	0	1 (4.5)	22 (13.7)		
Non adherence		0	0	8 (100)	0	0	8 (5.0)		
1 and 2		10 (38.0)	6 (23.1)	9 (34.6)	1 (3.8)	0	26 (16.1)		
all		1 (25.0)	3 (75.0)	0	0	0	4 (2.5)		

The association was assessed using the Pearson chi-square test, and *p* < 0.05 was considered to indicate statistical significance. HCPs, Healthcare providers; ADR, adverse drug reaction; MOH, Ministry of Health; FMHACA, Food, Medicine, Health Care Administrative and Control Authority; STG, standard treatment guideline; EPA, Ethiopian Pharmaceutical Association; DTC, Drug Therapeutic Committee.

### Attitudes of healthcare providers in the treatment of acute lymphoblastic leukemia

Regarding the attitudes of HCPs toward ALL treatment and ADR reporting, 108 (67.1%) respondents agreed that ADR reporting could benefit public health, while 76 (47.2%) stated that ADR reporting should be compulsory. Conversely, 33 (20.5%) and 34 (21.1%) respondents believed that ADR reporting is time-consuming with no significant outcomes and that awareness of parental socioeconomic status (SES) is crucial for ALL treatment outcomes, respectively.

A total of 57 (35.4%) HCPs agreed that thiopurine chemotherapy drugs reduce red blood cell, white blood cell, and platelet counts. Additionally, 49 (30.4%), 47 (29.2%), 67 (41.6%), and 55 (34.2%) respondents expressed beliefs that blood cancer is hereditary, TPMT variants alone do not explain all thiopurine toxicity, severe ADRs related to TPMT genetic variants have been identified, and there are ethnic differences in the TPMT gene, respectively. Furthermore, a minority of respondents, 19 (11.8%), correctly disagreed that thiopurine drugs are safe for pregnant women (Table 4).

**TABLE 4:** Attitudes toward adverse drug reactions (ADRs) associated with thiopurine use for the treatment of ALL among healthcare providers at the UoGCSH, Northwestern Ethiopia, 2023 (*n* = 161).

Items	Category	Profession							
		Physicians	Pharmacy	Nurse	Health officer	Midwives	Total	Pearson chi-square	P-value
		20 (12.4%)	19 (11. 8%)	105 (65.2%)	7 (4.3%)	10 (6.2%)	161 (100%)		
Do you feel that ADR reporting is part of their duty and can benefit the public health?								16.051	0.042
Agree		20 (18.5)	11 (10.2)	67 (62.0)	6 (5.6)	4 (3.7)	108 (67.1)		
Neutral		0	6 (15.0)	28 (70.6)	1(2.5)	5 (12.5)	40 (24.8)		
Disagree		0	2 (15.4)	10 (76.9)	0	1 (7.7)	13 (8.1)		
Do you agree thiopurine drugs are not good for pregnant women?								25.698	0.001
Agree		17 (24.6)	6 (8.7)	38 (55.1)	6 (8.7)	2 (2.9)	69 (42.9)		
Neutral		3 (4.1)	10 (13.7)	52(71.2)	1 (1.4)	7 (9.6)	73 (45.3)		
Disagree		0	3 (15.8)	15(78.9)	0	1 (5.3)	19 (11.8)		
Chemotherapy makes children only sicker and therefore cannot cure cancer?								22.501	0.004
Agree		10 (26.3)	5 (13.2)	19 (50.0)	4 (10.5)	0	38 (23.6)		
Neutral		2 (3.8)	5 (9.4)	37 (69.8)	3 (5.7)	6 (11.3)	53 (32.9)		
Disagree	8 (11.4)	9 (12.9)	49 (70.)	0	4 (5.7)	70 (43.5)			
Do you feel that thiopurine drugs prevent rejection after a solid organ transplant?								36.940	0.000
Agree		13 (27.1)	9 (18.8)	20 (41.7)	6 (12.5)	0	48 (29.8)		
Neutral		7 (7.4)	7 (7.4)	71 (75.5)	1 (1.1)	8 (8.5)	94 (58.4)		
Disagree		0	3 (15.8)	14 (73.7)	0	2(10.5)	19(11.8)		
Do you feel that reporting ADR should be compulsory?								23.678	0.003
Agree		16 (21.1)	10 (13.2)	42 (55.3)	6 (7.9)	2 (2.6)	76 (47.2)		
Neutral		4 (6.2)	4 (6.2)	50 (76.9)	1 (1.5)	6 (9.2)	65 (40.4)		
Disagree		0	5 (25)	13 (65.0)	0	2 (10.0)	20 (12.2)		
Do you feel that only ADR that cause persistent disability should be reported?								13.829	0.086
Agree		7 (11.5)	7 (11.5)	36 (59.)	6 (9.8)	5 (8.2)	61 (37.9)		
Neutral		7 (13.7)	4 (7.80	34 (66.70	1 (2.0)	5 (9.8)	51 (31.7)		
Disagree		6 (12.2)	8 (16.3)	35 (71.4)	0	0	49 (30.4)		
Do you feel that ADR reporting is a time-consuming activity with no outcome?								24.962	0.002
Agree		6 (18.2)	5 (15.2)	18 (54.5)	4 (12.1)	0	33 (20.5)		
Neutral		6 (9.0)	7 (10.4)	41 (61.2)	3 (4.5)	10(14.9)	67 (41.6)		
Disagree		8 (13.1)	7 (11.5)	46 (75.4)	0	0	61 (37.9)		
Do you believe Thiopurin drug ADR reporting is a professional obligation for all HCP?								21.935	0.005
Agree		14 (20.0)	4 (5.7)	47 (67.1)	5 (7.1)	0	70 (43.5)		
Neutral		4 (5.5)	11(15.1)	47 (64.4)	2(2.7)	9(12.3)	73 (45.3)		
Disagree		2 (11.1)	4 (22.2)	11 (61.1)	0	1(5.6)	18 (11.2)		
Do you feel that thiopurine drug chemotherapy lowers your amount of red blood cells, WBC, and platelets?								9.999	0.265
Agree		8 (14.0)	4 (7.0)	41 (71.9)	1 (1.8)	3 (5.3)	57 (35.4)		
Neutral		9 (12.3)	8 (11.0)	45 (61.6)	6 (8.2)	5 (6.80	73 (35.3)		
Disagree		3 (15.0)	7 (36.8)	19 (18.1)	0	2 (20.0)	31 (19.3)		
Do you feel TPMT variants alone do not account for all thiopurine drug toxicity?								16.313	0.038
Agree		7 (14.9)	7 (14.9)	25 (53.2)	6 (12.8)	2 (4.3)	47 (29.2)		
Neutral		12 (14.3)	8 (9.5)	57(67.9)	1 (1.2)	6 (7.1)	84 (52.2)		
Disagree		1 (3.3)	4 (13.3)	23 (76.7)	0	2 (6.7)	30 (18.6)		
Do you believe the identification of TPMT genetic variants that contribute to serious ADRs?								17.859	0.022
Agree		12 (17.9)	8 (11.9)	39 (58.2)	7 (10.4)	1 (1.5)	67 (41.6)		
Neutral		7 (9.1)	9 (11.7)	54 (70.1)	0	7 (9.1)	77 (47.8)		
Disagree		1 (5.9)	2 (11.8)	12 (70.6)	0	2 (11.8)	17 10.6)		
Do you feel an ethnic TPMT gene difference?								10.299	0.245
Agree		6 (10.9)	8 (14.5)	40 (72.7)	1(1.8)	0	55 (34.2)		
Neutral		12 (14.3)	8 (9.5)	51 (60.70	4 (4.80	9 (10.7)	84 (52.2)		
Disagree		2 (9.1)	3 (13.6)	14 (63.6)	2 (9.1)	1 (4.5)	22 (13.7)		
Do you think that blood cancer is a hereditary disease?								13.370	0.100
Agree		7 (14.3)	7 (14.3)	34 (69.4)	1 (2.0)	0	49 (30.4)		
Neutral		11 (14.7)	9 (20.0)	42 (56.0)	4 (5.3)	9 (12.0)	75 (46.6)		
Disagree		2 (5.4)	3 (8.1)	29 (78.4)	2 (5.4)	1 (2.7)	37 (23.0)		
Do you feel Awareness of parents SES is important for ALL treatment outcomes?								13.736	0.089
Agree		1 (2.9)	8 (23.5)	24 (70.6)	1 (2.9)	0	34 (21.1)		
Neutral		16 (15.4)	9 (8.7)	64 (61.5)	6 (5.8)	9 (8.7)	104 (64.6)		
Disagree		3 (13.0)	2 (8.7)	17 (73.9)	0	1 (4.3)	23 (14.3)		

*p* < 0.05 was considered to indicate statistical significance. TPMT, thiopurine methyltransferase; WBC, white blood cell; ALL, acute lymphoblastic leukemia; SES, socioeconomic status; UoGCSH, University Of Gondar Comprehensive Specialized Hospital.

### Practice of healthcare providers in the treatment of ALL and reporting ADRs

The present study revealed that only 13 (8.1%) patients experienced at least one thiopurine ADR in the past 12 months of clinical practice. Approximately 32 (19.9%), 50 (31.1%), and 58 (36.0%) HCPs reported that they always, usually, and sometimes provided their patients with appropriate advice on potential drug adverse effects, respectively. In contrast, 21 (13.0%) admitted they never advised their patients adequately regarding ADRs. Most respondents, 135 (85.7%), reported a lack of available ADR reporting forms. However, the majority of respondents, 109 (67.7%), expressed readiness to report any ADRs they might encounter in their future practice.

Few respondents, 44 (27.3%), believed that thiopurine medications induce remission in 50% of ALL cases. Additionally, 108 (67.1%) of the respondents were unsure whether TPMT genotype or TPMT enzyme activity testing was available in Ethiopia (Table 5).

**TABLE 5:** Thiopurine ADR reporting practices for ALL treatment among HCPs at the UoGCSH, Northwestern Ethiopia, 2023 (*n* = 161).

Profession											
Item		Category		Physicians	Pharmacy	Nurse	HO	Midwives	Total	Pearson chi-square	*p*-value
				20 (12.4%)	19 (11. 8%)	105 (65.2%)	7 (4.3%)	10 (6.2%)	161 (100%)		
Have you encountered patients with thiopurine ADR in the last12 months?										11.108	0.893
Yes			1 (7.7%)		4 (15.4%)	12 (69.2%)	0	1(7.7%)	13 (8.1)		
No			19 (12.8%)		15(11.5%)	93 (64.9%)	7 (4.7)	9(6.1)	148 (91.9)		
How many patients with Thiopurine ADR have you encountered?										25.962	0 .055
one			1 (25.0)		0	3 (75.0)	0	0	4 (2.5)		
Two			0		1 (16.7)	3 (50.0)	0	2 (33.3)	6 (3.7)		
Three			0		1 (11.1)	5 (55.6)	0	3 (33.3)	9 (5.6)		
Four			0		1 (33.3)	2 (66.70	0	0	3 (1.9)		
None			19 (13.7)		16 (11.5)	92 (66.2)	7 (5.0)	5 (3.6)	139 (86.3)		
I am very well prepared to report any ADRs notice in my future practice.									20.288	0.000	
Yes			20 (18.3)		12 (11.0)	67 (61.5)	7 (6.4)	3 (2.8)	109 (67.7)		
No			0		7 (13.5)	38 (73.1)	0	7 (13.5)	52 (32.3)		
Have you reported thiopurine ADRs?										2.192	0.701
Yes			0		1 (14.3)	6 (85.7)	0	0	7 (4.3)		
No			20 (13.0)		18 (11.7)	99 (64.3)	7 (4.5)	10 (6.5)	154 (95.7)		
Thiopurine medication-induced remission of ALL is?										24.966	0.070
100%			1 14.3)		0	5 (71.4)	0	1 (14.3)	7 (4.3)		
85%			7 (16.3)		8 (18.6)	27 (62.8)	1 (2.3)	0	43 (26.7)		
50%			4 (9.1)		4 (9.1)	27 (61.4)	1 (2.3)	8 (18.2)	44 (27.3)		
14%			6 (14.0)		5 (16.60	27 (62.8)	4 (9.3)	1 (2.3)	43 (26.7)		
4%			2 (8.3)		2 (8.3)	19 (79.2)	1 (4.2)	0	24 (14.9)		
Do you have ADR reporting forms?										7.888	0.096
Yes			6 (26.1)		4 (17.4)	13 (56.5)	0	0	23 (14.3)		
No			14 (10.1)		15 (10.9)	92 (66.7)	7 (5.1)	10 (7.2)	135 (85.7)		
How often do you advise patients on possible adverse effects of drugs?										59.825	0.000
Always			14 (43.8)		5 (15.6)	12 (37.5)	1 (3.1)	0	32 (19.9)		
Usually			4 (8.0)		8 (16.0)	26 (52.0)	6 (12.0)	6 (12.0)	50 (31.1)		
Some times			1 (1.7)		5 (8.6)	49 (84.5)	0	3 (5.2)	58 (36.0)		
Never			1 (4.8)		1 (4.8)	18 (85.7)	0	1 (4,8)	21 (13.0)		
Is TPMT genotype and TPMT enzyme activity tested in Ethiopia?										24.735	0.002
Yes			2 (7.4)		6 (22.2)	19 (70.4)	0	0	27 (16.8)		
No			5 (19.2)		7 (26.9)	10 (38.5)	0	4 (15.4)	26 (16.1)		
I don’t know			13 (12.0)		6 (5.6)	76 (70.4)	7 (6.5)	6 (5.6)	108 (67.1)		
How many pediatric oncology centers are available in Ethiopia?										60.981	0 .000
A = 2			1 (5.3)		2 (10.5)	15 (78.9)	0	1 (5.3)	19 (11.8)		
B = 4			3 (9.7)		2 (6.5)	25 (80.6)	0	1 (3.2)	31 (19.3)		
C = 10			3 (13.0)		4 (17.4)	12 (52.2)	0	4 (17.4)	23 (14.3)		
D = 5			3 (17.6)		2 (11.8)	10 (58.8)	0	2 (11.8)	17 (10.6)		
E = 3			7 (26.9)		4 (15.4)	8 (30.8)	7(26.9)	0	26 (16.1)		
I don’t know			3 (6.7)		5 (11.1)	35 (77.8)	0	2(4.4)	45 (28.0)		

Abbreviations: ALL, acute lymphoblastic leukemia, TPMT, thiopurine methyltransferase, UoGCSH, university of gondar comprehensive specialized hospital; ADR, adverse drug reaction, HO, health officer. The association was assessed using Pearson chi-square tests, and *P* < 0 .05 was considered to indicate statistical significance.

### The overall knowledge of HCPs

**FIGURE 1:**
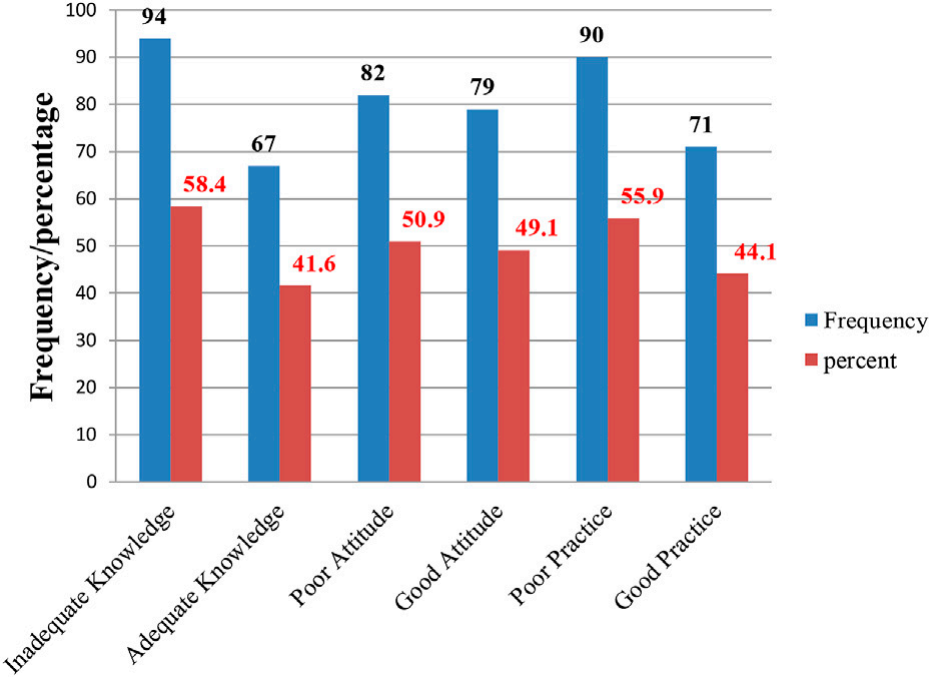
**The overall** knowledge, attitudes, and practices of HCPs toward ADR reporting regarding thiopurine drugs for the treatment of ALL at the UoGCSH, Northwest Ethiopia, in 2023 (*n* = 161).

The data presented in the chart strongly support the statement that, in general, the overall knowledge is inadequate. A significant 94% of respondents’ demonstrated inadequate knowledge, 82% exhibited a poor attitude, and 90% engaged in unfavorable or poor practices (Figure 1).

As shown in Table 6, following bivariate logistic regression analysis and multivariate binary logistic regression screening, only seven categorical variables were included in the final model. According to the final multivariable logistic regression model, the curability status of ALL was correlated with education level, clinical experience, profession, and gender. Additionally, comprehensive professional knowledge, chemotherapy knowledge, and training were found to be significantly associated with ADR reporting knowledge.

**TABLE 6:** Bivariate and multivariate logistic regression analysis of KAP at the University Of Gondar Comprehensive Specialized Hospital, Northwest Ethiopia, 2023 (*n* = 161).

Variables		Overall knowledge of HCP		COR (95% CI)	AOR (95%CI)	p.
		Adequate	Inadequate			P-value
Profession	Physicians	2 (2.9%)	1 8 (19.1%)	2.00	2.00	0.029
	Pharmacist	3 (4.4%)	16 (17.0%0	0.111 (0.016–0.755)	0.177 (0.024–1282)	
	Nurse	52(77.6%)	5 3 (56.3%)	0.187 (0.033–0.077)	0.210 (0.035–1262)	
	HO	5 (7.4%)	2 (2.1%)	0.981 (0.268–3.590)	0.998 (0.263–3795)	
	Midwives	5 (7.4%)	5 (5.3%)	2.500 (0.320–19.52)	2.18 (0.273–7.567)	
Have you trained on how to report ADR??	Yes	2 (2.9%)	2 3(24.4%)	1.00	1.00	0.020
	No	65 (97%)	71 (75.5%)	10.52 (2.388–46.415)	6.22 (1.328–9.206)	
Chemotherapy makes children only sicker and can’t cure cancer	Yes	9 (13.4%)	2 4 (25.5%)	1.00	1.00	0.039
	No	58 (86.5	70 (74.4%)	0.453 (0.195–1.050)	1.503 (0.587–3.851)	

Variables	Category	Is ALL curable?		COR (95%)	AOR (95%CI)	P-value
		Yes	No			
Gender	Female	28	41	1.00	1.00	0.241
	Male	36	56	1.062 (0.562–2.009)	1.041 (.523–2.071)	
Profession	Physicians	8	12	2.00	2.00	0.060
	Pharmacist	7	12	1.500 (0.325–6.918)	1.07 (0.184–6.213)	
	Nurse	40	65	1.714 (0.364–8.085)	0.098 (0.200–4.879)	
	HO	4	3	1.625 (0.443–5.967)	0.411(0.045–3.738)	
	Midwives	5	5	0.750 (0.107–5.238)	0.618 (0.079–4.80)	
Level of Education	Diploma	2	2	1.00	1.00	0.058
	Degree	47	71	1.511 (0.206–11.099)	1.653 (0.217–12.603)	
	MSC	8	14	1.750 (0.205–14.931)	2.298 (0.250–21.165)	
	MD	1	1	1.000 (0.034–29.807)	1.097 (0.025–48.331)	
	Specialist	6	9	1.500 (0.164–13.749)	1.257 (0.090–17.527)	
Clinical experience	≤3 year	9	20	1.00	1.00	0.292
	>3 year	55	77	0.630 (0.267–1.488)	0.582 (0.231–1.465	

As illustrated in Table 6, bivariate logistic regression analysis was conducted, where each independent variable was tested against the dependent variable, and variables with *p*-values less than or equal to 0.2 were included in the multiple logistic regression for further analysis. A *p*-value of less than 0.05 was considered statistically significant. According to the multiple logistic regression results, profession, training, and overall chemotherapy knowledge showed statistically significant associations with the overall knowledge of healthcare professionals (HCPs). Educational status also demonstrated a statistically significant association with the perceived curability of ALL.

Compared to physicians, pharmacists, nurses, and health officers, midwives were approximately 2.189 times more likely to possess overall knowledge of thiopurine drugs for the treatment of ALL-related ADRs [adjusted odds ratio (AOR): 2.189, 95% confidence interval (CI): 0.2731–7.567]. Healthcare professionals who had received prior ADR training were 6.227 times more likely to demonstrate adequate overall knowledge compared to those without any ADR training [AOR: 6.227, 95% CI: 1.328–29.206]. Furthermore, healthcare professionals with master’s degrees or specialisit were 2.298 and 1.257 times more likely, respectively, to exhibit good knowledge regarding the curability of ALL compared to those with diplomas or lower qualifications [AOR: 2.298, 95% CI: 0.250–21.165] and [AOR: 1.257, 95% CI: 0.090–17.527].

### Reasons for not reporting adverse drug reactions

Among the 161 study participants, 129 (80.1%) did not report ADRs, primarily due to the unavailability of reporting forms. Similarly, 136 (84.5%) respondents were uncertain about how to report ADRs, which further hindered their reporting practices. Additionally, 128 (79.5%) healthcare professionals refrained from reporting ADRs because of the absence of a responsible body monitoring ADRs in Ethiopia. Several participants cited multiple reasons for their failure to report ADRs (Table 2).

## Discussion

Thiopurines drugs are one of the cornerstones of current acute lymphoblastic leukemia (ALL) treatment. However, ADRs and drug toxicity can severely impact patient health, increasing morbidity, mortality, and hospitalization rates, thereby leading to unnecessary healthcare expenditures (Ray et al., 2022). To our knowledge, this is the first study to report and analyze the KAP of HCPs toward thiopurine drugs for the treatment of ALL in Ethiopia. Our study primarily targeted physicians, pharmacists, nurses, health officers, and midwives who closely interact with patients. Notably, nurses constitute the largest segment of this group, followed by physicians and pharmacists, which aligns with findings from a study conducted at Hiwot Fana Specialized University Hospital in Harar (Shanko and Abdela, 2018).

The findings of this study indicated that approximately 58.4%, 55.9%, and 50.9% of respondents demonstrated inadequate knowledge, unfavorable practices, and poor attitudes, respectively, toward thiopurine ADR treatments for ALL. However, thiopurine maintenance therapies are widely used globally (Toksvang et al., 2022), despite concerns such as myelotoxicity and hepatotoxicity limiting their utility (Guo et al., 2022). While genetic testing and TPMT enzymatic activity testing are available in developed countries for several TPMT variant alleles (Dean and Kane, 2020), these remain inaccessible in Ethiopia, with 65.1% of HCPs uncertain about their implementation.

Our study revealed that only a minority of respondents were familiar with terms such as “thiopurine drugs” 50 (31.1%), “TPMT gene” 22 (13.7%), “TPMT enzyme deficiency” 22 (13.7%), and “reporting.” Additionally, only 88 (54.7%) provided satisfactory responses regarding whom to prescribe thiopurine drugs, and 89 (55.3%) correctly identified thiopurine drug adverse reactions. Fifty point three percent (50.3%) of HCPs correctly identified **ALL** as the more prevalent condition, whereas 49.7% identified AML. Most studies indicate that ALL is the most prevalent hematological malignancy in children, accounting for approximately 86% of cases, with 91% of childhood leukemias being ALL and 9% being AML (Mostert et al., 2013; Kassahun et al., 2020).

Most studies report a high curability rate (98%) for ALL (Kassahun et al., 2020); however, our study found a widespread belief among HCPs that ALL is incurable. Awareness of curability rates varied: 15.1% knew the 98% curability rate, 28.9% believed it was 50%, 18.7% thought it was 25%, and 11.4% believed it was 4%, while 22.9% were unsure. This suggests inadequate training among HCPs in managing ALL with thiopurine drugs.

Thiopurine drugs, such as 6-mercaptopurine, exhibit variable toxicity due to polymorphisms in TPMT genes across ethnic groups (Rudin et al., 2017). However, our study contradicts this, as 50.6% of HCPs believed there were no ethnic differences in TPMT gene activity. Regarding thiopurine chemotherapeutic treatment, our study revealed that the knowledge levels of participants were very low (15.1%). These findings are consistent with a study conducted in India, where approximately 30% of participants were knowledgeable about chemotherapy (Yohannes, 2017).

Parental socioeconomic status (SES) significantly influences the decision to initiate childhood ALL treatment (86%), with better adherence observed among affluent parents (67%) (Mostert et al., 2013; Kassahun et al., 2020; Rudin et al., 2017; Yohannes, 2017; Mostert et al., 2008; Totadri et al., 2019). However, HCP awareness of SES’s impact on treatment outcomes was low, with only 20.5% demonstrating positive attitudes. Moreover, our study highlighted inadequate knowledge among HCPs regarding their professional obligation to report ADRs (39.1%), consistent with findings in Addis Ababa (21.1%) (Kefale et al., 2017).

Furthermore, a small proportion of respondents (23.0%) in our study were familiar with pharmacovigilance, similar to findings in the Jimma zone (19.5%) (Angamo et al., 2012; Adisa and Omitogun, 2019). Awareness of the national ADR reporting system (21.1%) and reporting forms (19.9%) was lower than reported in Lagos, Nigeria (40.4%) (Oshikoya and Awobusuyi, 2009). Additionally, only 20.5% and 39.1% of respondents knew that all HCPs are mandated to monitor ADRs in Ethiopia, likely due to limited training (15.5%) on ADRs and reporting.

Most HCPs (67.1%) agreed that ADR reporting is crucial for public health, aligning with findings in Addis Ababa (84%) (Kefale et al., 2017) but higher than in Jimma (57.31%) (Angamo et al., 2012). However, 20.5% perceived ADR reporting as time-consuming, potentially impacting their motivation (47.2%) to report ADRs in clinical practice.

Factors influencing ADR reporting knowledge included midwives, who were 2.189 times more likely to possess overall adequate knowledge compared to physicians, pharmacists, nurses, and health officers [adjusted odds ratio (AOR): 2.189, 95% confidence interval (CI): 0.2731–7.567]. Health professionals with previous ADR training were 6.227 times more likely to demonstrate overall adequate knowledge than those without [AOR: 6.227, 95% CI: 1.328–29.206]. Similarly, in one study, nurses (*p* = 0.002) and health officers (*p* = 0.018) had inadequate knowledge compared to physicians and pharmacists (Seid et al., 2018). In contrast, studies in the Philippines showed that nurses (86%), physicians (72%), and pharmacists (61%) had adequate ADR reporting knowledge (Carandang et al., 2015).

The table highlights a clear association between the healthcare profession and the level of knowledge regarding ADR reporting and related pharmacogenomic and pharmacovigilance concepts among healthcare providers at UoGCSH. Physicians and Pharmacy professionals generally possess significantly greater knowledge in these areas compared to Nurses, Health Officers, and Midwives. The statistically significant p-values for most knowledge-based questions indicate that these differences are unlikely to be due to chance. This suggests a need for targeted educational interventions and training programs tailored to the specific knowledge gaps identified within each professional group to improve ADR reporting practices and overall patient safety at the institution (Table 7).

**TABLE 7:** Association within the profession and knowledge of ADR reporting among healthcare providers at UoGCSH, Northwest Ethiopia, 2023 (*n* = 161).

Items	Category	Profession							
		Physicians	Pharmacy	Nurse	Health officer	Midwives	Total	Pearson chi-square	*p*-value
		20(12.4%)	19(11. 8%)	105(65%)	7(4.3%)	10(6.2%)	161(100%)		
Have you heard about the term thiopurine drugs?								0.461	0.000
Yes		70%	63%	22.9%	0%	0%	31.1%		
No		30.0%	36.8%	77.1%	100%	100%	68.9%		
Which is more prevalent in childhood blood cancer?								0.197	0.182
AML		40.0%	57.9%	48.6%	28.6%	80.0%	49.7%		
ALL		60.0%	42.1%	51.4%	71.4%	20.0%	50.0%		
Have you trained on how to report ADR?								0.378	0.000
Yes		50.0%	21.1%	9.5%	0%	10.0%	15.5%		
No		50.0%	78.9%	90.5%	100%	90.0%	84.5%		
Have you heard of the term TPMT gene?								0.413	0.0000
Yes		50.0%	15.8%	8.6%	0	0	13.7%		
No		50.0%	84.2%	91.4%	100%	100%	86.3%		
Have you heard of the term TPMT enzyme deficient?								0.265	0.023
Yes		35.0%	21.1%	9.5%	0.0%	10%	13.7%		
No		65.0%	78.9%	90.5%	100%	90%	86.3%		
Does chemotherapy make children only sicker and therefore cannot cure cancer.								0.153	0.440
Yes		30.0%	31.6%	18.1%	14.3%	10.0%	20.5%		
No		70.0%	68.4%	81.9%	85.7%	90.0%	79.5%		
Knowing the term pharmacovigilance?								0.472,	0.000
Yes		65.5%	47.4%	13.3%	0.0%	0.0%	23%		
No		35.0%	52.6%	85.7%	100%	100%	77.0%		
Knowing the national ADR reporting system?								0.413	0.000
Yes		60.0%	36.8%	13.3%	0.0%	10.0%	21.1%		
No		40.0%	63.2%	86.7%	100%	90.0%	78.9%		
Knowing the availability of ADR reporting forms?								0.433	0.000
Ye		60.0%	36.8%	10.5%	14.3%	10.0%	19,9%		
No		40.0%	63.2%	89.5%	85.7%	90.0%	80.1%		
Knowing the responsible body that monitors ADR in Ethiopia?								0.448	0.000
Ye		60.0%	42.1%	11.4%	0.0%	10.0%	20.5%		
No		40.0%	57.9%	88.6%	100%	90.0%	79.5%		
Which medications are appropriate for ALL types of blood cancers?								0.336	0.111
Mercaptopurine		65.0%	47.4%	35.2%	14.3%	30.0%	39.1%		
Thioguanine		20.0%	31.6%	30.5%	42.9%	20.0%	29.2%		
Azathiopurine		10.0%	15.8%	20.0%	42.9%	50.0%	21.1%		
I don’t know		5.0%	5.3%	14.3%	0.0%	0.0%	10.6%		

The associations were tested using the Pearson chi-square test (with values ranging between 0 and 1), and a significance level of *p* < 0.05 was considered statistically significant. ALL, acute lymphoblastic leukemia; UoGCSH, University of Gondar Comprehensive Specialized Hospital.

### Limitation of the study

This study has some inherent limitations. As a cross-sectional study, it does not establish causal relationships between healthcare professionals’ knowledge, attitude, and practice regarding adverse drug reaction reporting for thiopurine drugs. The use of self-administered questionnaires may have introduced recall and social desirability bias, potentially affecting the accuracy of responses. The study was conducted at a single center, the UoGCSH, which may limit the generalizability of the findings to other healthcare settings in Ethiopia or beyond. Future multi-center studies with a longitudinal design are recommended to address these limitations.

## Conclusion and recommendations

The majority of study participants demonstrated inadequate knowledge, unfavorable practices, and limited attitudes regarding thiopurine drugs, the ADR reporting system, and treatments for ALL. Additionally, most participants were unclear about the responsible body in Ethiopia for addressing ADR-related issues. Healthcare providers need to update their knowledge, improve communication skills, and provide accurate information to patients and caregivers.

To address these gaps, we recommend providing technical support and in-service training for HCPs, alongside implementing a robust reporting system. Such a system is fundamental to strengthening pharmacovigilance and enhancing both spontaneous and professional ADR reporting. Further studies are also needed to raise awareness among HCPs regarding childhood ALL curability, TPMT-related drug toxicity, ADRs, and the impact of parental socioeconomic status (SES) on treatment adherence. These efforts may ultimately improve adherence to childhood ALL treatment and enhance treatment outcomes.

## Data Availability

The original contributions presented in the study are included in the article/supplementary material, further inquiries can be directed to the corresponding author.

## Abbreviations

ADR, adverse drug reaction; ALL, acute lymphoblastic leukemia; DIC, Drug Information Center; DTC, Drug and Therapeutic Committee; EFDA, Ethiopian Food and Drug Administration; HCPs, Healthcare professionals; KAP, Knowledge, Attitude, and Practice; MOH, Ministry of Health; SPSS, Statistical Package for Social Sciences; STG, Standard Treatment Guidelines; TPM, Thiopurine mythyltransferase; UoGCSH, University of Gondar Comprehensive Specialized Hospital; WHO, World Health Organization.
